# Reviews and Book Notices

**Published:** 1878-05

**Authors:** 


### Reviews and Book Notices.

SPINAL DISEASE AND SPINAL CURVATURE; Their treatment by suspension and the use of plaster of Paris bandage. By Lewis A. Sayre, M. D., etc., etc.: London, Smith, Elder & Co.; Philadelphia, J. B. Lippincott & Co., 1878.

This little volume of 121 pages, dedicated to the British physicians and surgeons who accorded such a generous reception to the author during his late visit to England, has the merit of presenting in a convenient form full directions respecting the mode of application of the plaster-jacket in spinal curvature. It is embellished with eighteen photographs and seventy wood cuts, which not only serve to illustrate the principles to be observed in the application of this dressing, but also the practical results obtained by its use in the case of patients. Many of the cuts are those which have already appeared in the author's recently published lectures on Orthopedic Surgery.

It is needless, at this late day, to explain to our readers Professor Sayre's method of enveloping the trunk in a shell constituted of plaster and "cross-barred wiggin,"—often reinforced by vertical strips of perforated tin or other metal—as a result of which support is obtained that relieves the articular faces of the vertebræ in Potts' disease from the injurious results of pressure. Still less, perhaps, is it necessary to observe that the benefits obtained by the adoption of this method of treatment, have exceeded the most sanguine hopes of those who have employed it. The numerous papers which have appeared in the medical journals, Prof. Sayre's public demonstrations of its utility, as well as his practical exposition of the subject in his lectures on Orthopedic Surgery, and his report published (and reprinted) in the Transactions of the American Medical Association for 1877, have fully satisfied the demand for clear and definite information on the subject.

We do not propose to discuss the attempts which have been made to detract from the reputation of the author by charging that he was not the first to suspend a patient and then to completely surround the body with a plaster cast. Very recently, and, unless we are mistaken, since the author's return to this country, the attempt of this character on the part of Dr. Bryan, of Kentucky, has been succeeded by another in behalf of some German practitioner, who has long used the identical swing employed by Prof. Sayre.

There should be, at this day, few inventors and discoverers who are not fully prepared to encounter charges of this sort. There is nothing new under the sun; and in point of fact, no man ever really originates anything. The history of the discovery of anæsthesia is a striking commentary upon the fact that when men's minds are generally intent upon a problem, several individuals will simultaneously so nearly reach the solution, that it is well nigh impossible to adjudicate between their rival claims.

We are only surprised, in this present matter, that a man so robust, physically and mentally, as our author, should have thought it worth his while to take up the cudgels in self-defense on the question of priority. To the professional world it is a matter of very small moment indeed, whether Dr. Bryan first suspended his patient from the gas pipes of Bellevue Hospital, or whether some other hospital interne preceded him in doing this. All the honor, all the credit, all the immense prestige to be gained from a quick and clear appreciation of the value of encasing the entire trunk in plaster, of giving public demonstrations of its practical value, of clearly and vigorously presenting its claims to the profession and the world at large, and of finally placing the method in the high position of a received and accepted procedure—all this belongs justly and legally to Prof. Sayre. It is his, and no man can take it from him. He deserves every word of the whole-hearted encomiums of his professional friends in England, many of which were uttered with the mute commentary of tears. His name will always be inseparably associated, the world over, with the plaster of Paris jacket, all critics, rivals, claimants and even enemies to the contrary notwithstanding. We believe it is not exaggeration to say that the honor is one in which every American surgeon may feel an honest pride.

J. N. H.

### Transactions of State Medical Societies.

1. *Transactions of the Medical Society of the State of Pennsylvania*, at its twenty-eighth annual session, held at Harrisburg, June 13, 1877, pp. 428.
2. *Transactions of the Twenty-second Annual Meeting of the Ohio State Medical Society*, held at Put-in-Bay, June 12th, 13th and 14th, 1877, pp. 200.
3. *Transactions of the Eighth Annual Session of the Medical Society of Virginia*, held in Petersburg, October 23d, 24th and 25th, 1877, pp. 192.
4. *Transactions of the Canada Medical Association*, tenth annual meeting, Montreal, Sept. 12th and 13th, 1877, pp. 244.

1. The larger portion of the *Transactions of the Pennsylvania Society* is made up of reports from the county medical societies on topography, meteorology, mortality, and prevalent diseases in the different counties.

Dr. P. D. Keyser presented the result of his examinations of eyes affected with conjunctivitis and blepharitis ciliaris, in regard to the presence of refractive errors. Finding either hypermetropia or astigmatism in every case, he concludes that ametropia is a very common cause of these inflammations of the eye.

Dr. Benjamin Lee takes occasion to make some very instructive remarks on the *diagnosis of psoas abscess* in connection with the history of a patient who had a well-developed psoas abscess, while "none of the numerous physicians who had examined him had suspected its existence." The patient was a well-developed, thick-set boy of ten years. "When four years old he was attacked with whooping-cough. From this time forward his parents noticed an alteration in his gait and carriage. He was awkward in his movements and could not run like other children. At two different times he had attacks of severe pain originating in the left side and shoulder and extending around to the spine." In August, 1874, he fell from a hay-mow, the distance was about fourteen feet, and the brunt of the blow was borne by the left ischium. "Within a month after, he was attacked with acute pain in the left side of the abdomen, in the region of the descending colon. The agony was so intense that he was held on the lap for a week, the only relief being from strong pressure with the hand over the seat of pain." He was treated for "inflammation of the colon." The attack subsided, but a month later it was noticed that his spine was curved to one side. This was pronounced by a surgeon to be "a *trifling* muscular contraction," and the use of Indian clubs and dumb-bells, exercise and fresh air were advised. This was on the day previous to Dr. Lee's first visit. The condition of the patient as found by the doctor, may best be described in his own words: "When I entered the room, the patient was lying, dressed, upon a bed. I observed first, that he rose with great difficulty, turning on to the side before attempting to sit up, and holding his shoulders quite stiff. I requested him to walk, with his clothing still on, and found that he stooped forward considerably, keeping the knees bent, the heels flat on the floor, the left toe pointed in, and the buttocks projected much posteriorly, especially on the left side. He complained of no pain on walking, and could even step down heavily with either or both feet without apprehension. There was a tendency to rest the left hand on the knee of that side. I now directed him to strip, and examined him first standing. I found that when he straightened out the right leg, the left foot was raised from the floor, the hip being much and the knee slightly flexed. The spine was curved forward deeply in the lumbar region, as in lordosis, but without any projection higher up. It was also curved to the right, throwthe shoulders towards the left, and depressing that of the left side. I now made him sit down, when, presto! every particle of spinal curvature vanished, and in place of the lordosis, there was the slightest possible bulging of the lumbar spine backward, and a certain rigidity in the prominent tract. It was evident, therefore, that the contraction producing the antero-lateral curvature was not in the muscles of the dorsum of the spine and trunk, but in those which flex the thigh on the pelvis, or *vice versa*."

These muscles are, on the one hand, the proper *flexors* of the thigh, or, on the other hand, the *psoas* with the *iliacus*. Dr. Lee decided against the flexors, "because the trunk was flexed on the thigh, rather than the thigh upon the trunk; in other words, he bent the body forward rather than drew the limbs up;" and from the absence of the characteristic signs of disease of the hip-joint, which contractions of the flexors generally would indicate. The contraction was therefore located in the *psoas* muscle. The patient was caused to lie down on the floor on the flat of his back; and a careful pressure directly above the ramus of the pubes on each side of the abdomen, met on the left side with "a firmly resistant surface, about level with the brim." "This tumor was smooth and even, elastic—under firm pressure presenting a sense of deep-seated fluctuation—and rounding off gradually at its upper end." The presence of a psoas abscess was therefore, demonstrated, "caused immediately by the severe fall alluded to, but primarily by the succussion of the vertebræ and cartilages years before, by the paroxysms of whooping-cough."

The boy was supplied with a well-fitted spinal splint, a spinal swing, and a trapeze, in the use of which he was instructed, and returned home. In November he came back to Philadelphia, with "a well-defined tense tumor of considerable size, below Poupart's ligament." The aspiration was only partially successful, and an injection of salicylic acid and iodine for setting up a healthy action in the cavity, failed entirely. The pus rapidly re-accumulated and made its way along the track of the needle, and the abscess soon discharged freely. Hectic set in with night sweats; but when the patient was removed from his dark, gloomy, and very small room, to a place where he could have sunshine, fresh air, and homely diet, he began to improve so quickly, that "a month after the spontaneous discharge of the abscess, he left for home greatly improved." The systematic use of the swing was recommended, and the wheel crutch added, in order to restore the use of his limbs. "During all this time he was never, *day or night*, without his spinal splint." In May of last year, the patient was reported as "stronger than he ever was, in perfect health; his back entirely straight; taking the most violent exercises, and never complaining of pain, weakness, or soreness."

Dr. JOHN H. PACKARD found the elastic ligature very useful in the treatment of urethral fistulæ. In a case with three fistulous openings at the perineo-scrotal region, perineal section having been performed, an elastic ligature was drawn through each fistula, and brought through the wound of operation, tightened so as to produce slight constriction, and its ends tied together with a thread. The constriction was renewed every two or three days, as the tissues yielded; they closed up behind the ligature very kindly as it made its way through, and in a few weeks the fistulæ were divided and healed.

The "new" *modification of Syme's operation*, which Dr. W. R. HAMILTON describes, was first suggested by the late Professor Linhart, of Würzburg, in 1856, and has, ever since, been taught and practiced in all medical schools of the European continent.

2. The less voluminous *Transactions of the Ohio Society*, contain a report by Dr. S. S. GRAY, of Piqua, on the so-called *Milk-sickness* in animals. "If left alone, the sick animals stand or lie around for a few days, refusing to eat, and dying soon. But if exercised violently, they are seized with trembling, and in many cases fall down and die suddenly." "The milch-cow is comparatively safe, the poison being eliminated from her system in the milk." And through such milk the disease is spread among other animals; it is communicated to men by the milk and the flesh of diseased animals. The symptoms are languor, variable appetite, gnawing at the stomach, nausea, torpidity of the bowels, stiffness of the limbs, an aversion to exercise, an insatiable thirst and great restlessness. Breathing, slow, with occasional sighing; tongue moist and but slightly coated, pulse normal. The nature of the poison, which when introduced into the stomach with the food, produces these symptoms, is still an unsettled question. It originates only in wild and uncultivated lands; cultivation destroys it.

Dr. J. H. POOLEY, of Columbus, reported four cases of *peri-*

*typhlitic abscess* successfully treated by incisions. The idea of cutting down into a perityphlitic abscess, was first acted upon by Hancock, of London, in 1848. But though the operation was entirely successful, no one followed his example until in 1867, Dr. Willard Parker, of New York, recalled the attention of the profession to the subject, by the publication of some successful cases. Since that time the operation has often been performed, and is now recognized as legitimate surgical treatment. Dr. Pooley could collect fifty cases with only four deaths.

Dr. JONATHAN MORRIS, of Ironton, adds his testimony in favor of *chloral hydrate as a remedial agent in puerperal convulsions*, in a report of two cases of this kind, in which it was administered with the best result.

3. The *Transactions of the Virginia Society* are published in the January number of the *Virginia Medical Monthly*. The advantages of this plan, over the issuing of a volume of transactions, are so obvious that other societies will probably be quick to follow the "new departure" of Virginia. The addresses, which fill over one-half of the transactions, are interesting retrospects of the recent advances in obstetrics, practice of medicine, hygiene and public health.

DR. J. S. WELLFORD, of Richmond, reports a series of cases of *poisoning by custards and ice creams*. A few hours after partaking of custard pie, not quite fresh, persons were taken sick with vomiting, purging, pain in the bowels, cramps in arms and legs, and general prostration. A boy of two years died ten hours after he was taken sick. The autopsy revealed inflammation of the gastric and intestinal mucous membrane, but no erosions or ulcerations. The chemical analysis, "found sufficient mercury in that portion of the contents of the stomach analyzed, which if it existed in the form of corrosive sublimate, would amount to from 0.35 to 0.5 of a grain, sufficient to cause death." The coroner's jury gave the verdict, "poisoned by mercury," with which opinion Dr. W could not agree; he thought that the custard, which was described by the patient as "bitter, sour and unpleasant to the taste," had undergone some decomposition in its albuminoid articles, which caused the irritation and inflammation of the intestines.

Dr. Martin L. James, of Richmond, contributed an interesting paper on *heart-clots*, reporting three cases which had fallen under his observation. The first patient was a woman of forty-three years; for several years she had been suffering from indigestion, frequent spells of diarrhœa, and congestion of the liver, and had become very anæmic and emaciated. But all this time, frequent and careful examinations had found all the organs and functions, other than the digestive and nutritive, in perfect health. On the 18th of January, 1871, a season of the most distressing nausea supervened, continuing for several days, and to a greater or less extent to the close of life, which occurred eight days from that time. During the night of the earlier portion of this season of nausea, the most remarkable cardiac murmur was developed. "It was heard over every portion of the chest, front and rear, and distinctly at a distance of four feet from the bed, the patient herself and all of the attendants hearing it. It was a constant vibratory and somewhat musical murmur, heard with the first sound of the heart, or to speak more properly, supplanting the first sound of the heart, highest pitched at its earliest portion, somewhat resembling the *frémissement cataire* of the French, but resembling to a striking degree the chirping of a young chicken. * * When, however, the ear was applied closely to the chest, it was found to be blended with a bellows murmur, of some roughness of tone. It continued uninterruptedly to the time of the patient's death, though toward the close of life, as the heart became enfeebled, it was heard with less intensity." The autopsy showed the structure of the heart in all respects normal; but a flattened fibrinous clot, of considerable firmness at the time of its removal, white in color, with a slight shade of buff, and homogeneous in structure, was found extending from the apendix auriculæ of the right sight, intertwined with the musculi pectinati running through the auriculo-ventricular opening into the ventricle, where it became entangled with the chordæ tendineæ and columnæ carneæ, being altogether about five inches in length, one inch and a quarter wide, and three lines in thickness.

The other two cases gave a similar history; the presence of heart-clots was diagnosticated from the presence of that peculiar chirping murmur, and the diagnosis was, in both cases, confirmed by the autopsy.

Dr. Wm. Selden, of Norfolk, reports four cases of intra-capsular fracture of the femur, which resulted in bony union.

The value of *iodoform* as a local application in syphilitic, scrofulous, and indolent ulcers, is demonstrated by Dr. J. E. Chancellor in a transcript of some cases.

4. In the *Canadian Transactions*, a typical case of Addison's disease is reported by Dr. Geo. Ross. The autopsy found all organs in a normal condition except the supra-renal capsules. These are enlarged, nodular, and firm to the touch. On section they show several larger and smaller caseous nodules.

Dr. Joseph Workman expresses his surprise that *acetate of lead in large doses* is not oftener employed in severe post-partum and other hemorrhages. Given "several drachms in the course of a few hours," it is the most efficient hæmostatic, and its use was never followed by any evil constitutional results.

Dr. Ad. Alt has examined a dozen *epithelial tumors of the anterior third of the eye-ball.* In regard to epithelial growths of the cornea, he states that they "do not seem to originate indiscriminately at any part of the corneal tissue, but only at the corneo-scleral junction." The first evidence is an excessive cell-formation in the epithelial layer of the conjunctiva or cornea. "This hyperplasia, acting like a foreign substance, keeps up a constant irritation of the underlying tissue, which leads to inflammation and destruction of the latter, and enables the epithelial cells to invade it. The epithelium grows into the mother-tissue in the shape of cylinders, which, by branching off form secondary and tertiary cylinders, and so on."

Considerable diversity of opinion exists at the present time as to the justifiableness of the operation of *excision of the knee joint.* Dr. Geo. E. Fenwick puts in a good word in favor of the operation, and sustains his opinion by the report of thirteen cases which were operated upon in the Montreal general hospital between 1865 and 1877, with the following result: Cured, 9; doubtful, 1; died of pyæmia, 1; amputated, 2.

F. C. H.

Cerebral Hyperæmia. By William A. Hammond, M. D., late Surgeon-General U. S. army; Professor of Diseases of the Mind and Nervous System in the Medical Department of the University of the city of New York, and in the Medical Department of the University of Vermont; Fellow of the College of Physicians of Philadelphia, and of the Academy of Arts and Sciences, Boston; Member of the Academy of the Natural Sciences of Philadelphia; of the American Philosophical Society; of the New York Neurological Society; of the American Neurological Association; Corresponding Member of the British Medical Association; Member of the Medico-chirurgical Society of Edinburgh, etc., etc. New York: G. P. Putnam's Sons, 182 Fifth avenue. 1878.

The pamphlet thus indicated, is an expansion of an essay read not long since before the New York Neurological Society. It must be confessed that, considering the immense array of titles adorning the title page, the work itself does little to justify the reputation of its author. Instead of a clear, sharp picture of cerebral hyperæmia, we are treated to a confused and hazy panorama of symptoms, which may be occasioned, many or all of them, by a dozen different conditions of physical disorder; and when the reader begins to look solicitously for some fixed point upon which to plant himself, he is blandly assured by the insinuation that, though it may be difficult for the inexperienced to distinguish the truth among so many contradictions, it is easy enough for the skillful. There is, also, a great and unnecessary parade of learning—an almost garrulous wandering at random over the whole field of cerebral pathology—which, though utterly useless to the student or practitioner who may read, cannot be deemed wholly without value if this were a book to slip into the hands of such of the laity as might need to be impressed with the profundity of the learning of its author. In fact, it is doubtful whether such people could peruse many of its pages without being incontinently filled with the belief that cerebral hyperæmia is the one precise and all sufficient cause of all their little aches and pains; and that their ordinary physician, who hints at cigars, wine, improper meals and their like, is most decidedly an old fogy of the worst kind. On the other hand, it will be equally impossible for that same old fogy to repress a growing suspicion that the work in question, is the offspring of a brain in which cortical degeneration is gradually taking the place of cerebral hyperæmia. H. M. L.

TRANSACTIONS OF THE NEW YORK PATHOLOGICAL SOCIETY. Volume second. Based on the proceedings of the year 1875, and largely supplemented from the records of 1844 to 1877. Edited by John C. Peters, M. D., President of the Medical Society of the county of New York. New York: Printed for the society by Wm. Wood & Co. 1877.

This volume of nearly 200 pages, is made up of reports on pathological processes in the organs of the abdominal cavity. It contains the reports of 208 cases of diseases of the intestines; 23 cases of malignant diseases of the peritoneum; 13 cases of diseases of the pancreas; and 58 cases of diseases of the liver.

The committee on publication and the editors, wished to make each volume of these transactions "a valuable book of reference in most of the great emergencies of medical and surgical practice, and also in the recognition of obscure cases." They have ably acquitted themselves of their task in the present volume. The systematic order in which reports on diseases of the same organs, and records of similar cases are grouped together, is well adapted to the great purpose the editors aimed at, making the consultation of the work easy, attractive, and instructive.

INJURIES OF THE EYE AND THEIR MEDICO-LEGAL ASPECTS. By Ferdinand Von Arlt, M. D., Prof. of Ophthalmology in the Imperial University, Vienna. Translated by Dr. Chas. S. Turnbull, M. D., etc. Philadelphia: Claxton, Remsen & Haffelinger. 1878.

Whatever is written by so eminent a man and close observer as Prof. Arlt, is always worthy a most careful study. The present work is intended more for the surgical practitioner than the physician or student. It was originally published in separate articles in the *Wiener Medicinische Wochenschrift*, and the very favorable notices which they received, and the great demand for them, caused the author to publish them in book form. He divides his subject into three classes:

1st. Injuries produced by sudden compressson or concussion of the eye.

2d. Injuries produced by the entrance of a foreign body not acting chemically.

3d. Scalds and corrosions of the eye ball.

He also devotes one chapter to simulated diseases. A class of diseases very rarely seen by American practitioners. The translating and printing are creditably done. L. W.

